# To Squeeze or Not to Squeeze: A District General Hospital Review of Knee Arthroscopy With and Without Tourniquet

**DOI:** 10.7759/cureus.84154

**Published:** 2025-05-15

**Authors:** Zeeshan A Khan, Muhammad Abdullah, Sadaf Altaf, Wiqqas Jamil

**Affiliations:** 1 Orthopaedics and Trauma, Manchester Royal Infirmary, Manchester, GBR; 2 General and Colorectal Surgery, Manchester Royal Infirmary, Manchester, GBR; 3 Medicine, Tameside General Hospital, Manchester, GBR; 4 Orthopaedics and Trauma, Tameside General Hospital, Manchester, GBR

**Keywords:** arthroscopy knee, knee arthroscopy (ka), operative outcomes, pain, postoperatve analgesia, tourniquet

## Abstract

Background

Knee arthroscopy (KA) is a frequent technique used to treat knee joint problems across the world. Tourniquets are often utilised in KA; however, their safety is still debated. The aim of this study was to determine postoperative pain, recovery time, and postoperative surgery analgesia in patients undergoing KA with and without a tourniquet.

Methods

This prospective cohort study was carried out in the Orthopaedic Department of a district general hospital. A total of 28 patients undergoing KA were included and divided into two groups (14 tourniquet and 14 no-tourniquet). Pain, time for recovery, and use of opioid analgesia were recorded. Minimal clinically important difference (MCID) was also calculated to review the relevance of the difference in pain scores at different intervals.

Results

There were 23 (82.1%) males and five (17.9%) females from a total of 28. There was no significant difference (p=0.149) in mean ages of the patients in the tourniquet group (28.36±7.52 years) and the non-tourniquet group (29.57±5.61 years). The tourniquet group experienced a mean postoperative pain of 8.00±1.04 on the Visual Analog Scale, while the other group reported 8.21±0.89 (p<0.381). This reduced to 1.14±1.027 in the tourniquet and 0.29±0.469 in the non-tourniquet group (p=0.00) at six weeks' follow-up, thus indicating higher postoperative pain in the tourniquet group. This difference was also above the threshold for MCID, suggesting that it was clinically relevant and meaningful from a patient perspective, too. A greater requirement for postoperative surgery analgesia was also observed in the tourniquet group; however, the difference was not statistically significant.

Conclusion

Knee arthroscopy surgery without a tourniquet leads to lower pain and relatively lower analgesic demand postoperatively. Future studies should aim to include a large sample size and long-term functional outcomes for a better understanding of the topic.

## Introduction

Knee arthroscopy (KA) is one of the most commonly performed orthopaedic treatments on a global scale, for a range of knee joint pathologies [1]. When conventional approaches prove to be inadequate, KA is often chosen and executed as a medical intervention [2]. Haemorrhage-induced reduced visibility of anatomical features during surgical procedures may result in iatrogenic damage and misalignment of implants [3]. As minimally invasive operations like arthroscopy become more common, tourniquets are utilised to reduce bleeding and improve tissue visualisation. The tourniquet was invented by Jean Louis Petit, a derivative of the French 'tourner', meaning "to turn" in 1718 [4]. A tourniquet is utilised in the vast majority of KA to maintain the operative area sterile and to avoid excessive blood loss [5]. The use of a tourniquet during KA is linked to a number of potentially fatal consequences, including reperfusion syndrome, variations in metabolism and temperature, thrombosis, cardio-respiratory decompensation, and arrest with exsanguination [6].

Many orthopaedic units still routinely use a tourniquet during soft tissue procedures in knee arthroscopy, likely believing that a clear operative view can only be achieved with one [7]. Numerous studies have shown that using a tourniquet for the procedures increases the likelihood of complications such as limb ischaemia [8], deep vein thrombosis (DVT) [9], pain [10], nerve damage [11], and muscle weakness [12]. Further evidence suggests that tourniquet use has an influence on postoperative recovery. While the application of a tourniquet is associated with higher postoperative pain after various surgeries, its significance in this area is not entirely apparent, with some studies finding an increase in pain if it is not used [13].

Tourniquets are used by most surgeons during arthroscopic operations to increase their field of vision [14]. However, patients experience postoperative pain, necessitating greater doses of analgesics and a longer length of recovery time before they may be released. This has raised uncertainty regarding the use of a tourniquet for routine knee arthroscopic procedures. 

The primary objective of this study was to assess the impact of tourniquet use on postoperative pain. The secondary objectives included assessing the impact of tourniquet use on time spent in hospital and opioid analgesia requirement postoperatively.

## Materials and methods

This prospective cohort study was carried out in the Trauma and Orthopaedic Department of a district general hospital over a six-month period. Patients undergoing knee arthroscopy were identified using the electronic record.

Specific eligibility criteria were set, and eligible participants included adults over the age of 18, of any gender, with a confirmed meniscal injury radiologically on MRI scan, provided they did not have severe osteoarthritis requiring meniscal debridement. Patients with concurrent meniscal and anterior cruciate ligament (ACL) damage requiring surgical intervention and who lost follow-up or had missing data were excluded from the study. All participants in the study provided written informed consent. 

Patients were allocated one of two treatment categories by a sequence of letters: A referring to tourniquet-assisted (n=14) and B referring to non-tourniquet (n=14) arthroscopy using sequentially numbered, opaque, sealed envelopes. The allocation sequence and preparation of the concealed envelopes were completed by a third person who was not involved in the conduct of the study. This was concealed from the participants, surgeon, and statistician. Group A included patients who underwent KA with a tourniquet (34 inches, single bladder, dual port, Zimmer) around the thigh. Tourniquet pressure was set to a maximum of 300 mmHg. It depended on the systolic blood pressure of the patient. The pressure was kept 100 mmHg above systolic pressure for a duration of 90-120 minutes. Group B included those who underwent KA without the tourniquet. All operations were carried out by the same consultant surgeon to ensure standardisation.

Pain was evaluated using the Visual Analogue Scale (VAS) before surgery, immediately postoperatively, and at two- and six-week follow-up visits. Patients were carefully instructed on the use of the scale, which ranged from zero, indicating no pain, to 10, representing the worst pain imaginable. Data collection included recording VAS scores at each time point as well as documenting postoperative opioid consumption. Postoperative analgesia was standardised with oral morphine (10 mg Oromorph) for all patients due to its ease of administration and provision of strong opioid analgesia. Intravenous opioids were not used. Although NSAIDs may have been administered as part of routine care, their use was not systematically recorded, and therefore, opioid consumption mentioned as "1" represented 10 mg Oramorph. The timings of discharge from the hospital and the recovery area were also recorded. Quantitative variables such as age and VAS are presented as mean and standard deviation. Qualitative variables such as gender are presented as frequencies and percentages. An independent t-test was used to compare mean pain, mean time in recovery area, mean time in day surgery unit and mean opioid consumption postoperatively in both groups.

A p-value of <0.05 was considered statistically significant. To explore the clinical significance of observed differences between the tourniquet and non-tourniquet groups, we calculated the minimal clinically important difference (MCID) using the 0.5 standard deviation (SD) approach. This distribution-based method provides a widely accepted estimate of the smallest change in a score that patients are likely to perceive as beneficial, particularly when anchor-based data are unavailable. MCID values were calculated for both groups at each postoperative time point by taking half of the corresponding group's standard deviation. The data was statistically analysed using SPSS version 21 (IBM Inc., Armonk, New York). 

## Results

There were 23 (82.1%) males and five (17.9%) females out of 28 patients. All 28 patients were followed up at six weeks. Table 1 summarises the pain level at different time intervals. The mean pain on baseline was 8.00±1.04 in the tourniquet group and 8.21±0.89 in the non-tourniquet group; however, this difference was not statistically significant. Pain two hours post-surgery was lower in the non-tourniquet group, with this difference being statistically significant (p=0.042). At six weeks' follow-up, the mean pain was greater in the tourniquet group than in the non-tourniquet group, with a statistically significant difference (p=0.00). In the tourniquet group, MCID estimates ranged from 0.22 to 0.52, while in the non-tourniquet group, they ranged from 0.23 to 0.60. 

**Table 1:** Mean pain score on pain VAS at different time intervals between tourniquet and non-tourniquet groups VAS - Visual Analogue Scale

Time interval	Group		p-value	t-test	95% confidence interval	
	Tourniquet	Non-tourniquet			Lower	Upper
At baseline	8.00±1.04	8.21±0.89	0.381	-0.586	-0.966	0.538
1 hour after surgery	6.71±.825	6.29±1.204	0.423	1.089	-0.374	1.231
2 hours after surgery	5.43±.514	5.07±.475	0.042	1.911	-0.027	0.741
At discharge	3.64±0.633	3.14±.770	0.659	1.876	-0.048	1.048
At 2 weeks follow-up	2.21±0.43	1.93±0.73	0.189	1.265	-0.179	0.750
At 6 weeks follow-up	1.14±1.027	0.29±0.469	0.000	2.841	0.237	1.477

Figure 2 represents the mean pain score between the two groups as a linear graph over the six-week period postoperatively.

**Figure 1:**
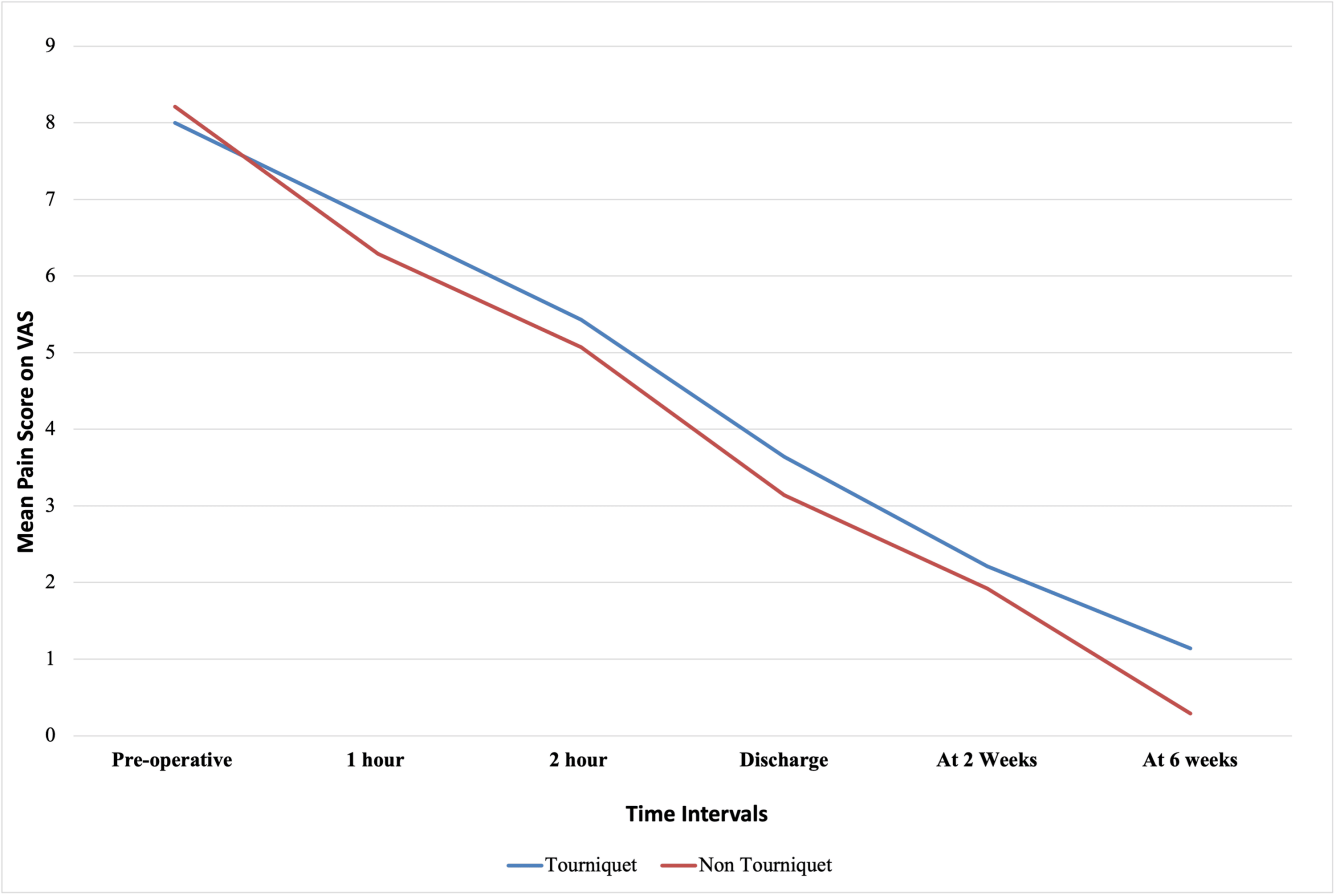
Represents a line graph of mean pain score on VAS at different intervals in the tourniquet (Blue) and non-tourniquet (Red) groups VAS - Visual Analogue Scale

The mean time spent in the recovery area and day surgery unit was shorter in the non-tourniquet group, however, this difference was not statistically significant. Usage of postoperative opioids was also lower in the non-tourniquet group, but not statistically significant, than in the other group. Table 2 represents the data.

**Table 2:** Mean time spent in the recovery area, the day surgery unit, and the number of times opioid analgesia was requested

Variable	Tourniquet	Non-tourniquet	p-value	t-test	95% confidence interval	
					Lower	Upper
Mean time in recovery area (minutes)	48.21±5.07	45.14±4.34	0.564	1.656	-0.741	6.884
Time in day surgery unit (hours)	38.00± 5.084	34.79±4.80	0.602	1.719	-0.629	7.058
Postoperative opioid analgesia	1.120± 0.28	1.09±0.23	0.069	1.101	-0.092	0.307

## Discussion

As less-invasive techniques have developed, KA is among the most common orthopaedic procedures carried out globally. There has been discussion of using a tourniquet to lessen the quantity of blood lost during this procedure. Since its introduction in 1904, the tourniquet has been a staple in the field of extremity surgery [15]. Similar to any other surgical procedure, using a tourniquet carries some risks. This is why the use of a tourniquet in routine KA surgery has never been conclusively answered. Many clinical studies, including randomised controlled trials (RCTs), have been performed to ascertain which option is better, but the outcomes have not always been consistent [16-18]. An RCT done by Tibrewal et al. showed that routine KA, which necessitates the insertion of a tourniquet, does not considerably improve the patients' overall quality of life or functional outcome [19]

Nowadays, during a traditional KA, various surgeons make varying decisions on tourniquet use. Our study included 28 individuals who had KA. A total of 21 patients (82.1%) were male and five (17.9%) were female. Relatively higher age of patients was noted to be in the non-tourniquet group (29.57±5.61) as compared to the patients in the tourniquet group (28.36±7.52). In several additional studies, a larger proportion of male patients undergoing knee surgery has also been recorded. For example, a study done by Reda et al. included 49 males and only nine females, with a mean age of 25.5 among patients receiving ACL surgery [10]. Findings from our study were also consistent with this study's findings, as described previously.

Our results showed that baseline pain was 8.00 in the tourniquet and 8.21 in the non-tourniquet group. According to the study's results, mean pain was higher in the tourniquet group than in the non-tourniquet group at all intervals postoperatively. The difference in pain at six weeks follow-up between the two groups in our study, with pain being lower in the non-tourniquet group, was not only statistically significant but also of clinical relevance. At six weeks postoperatively, the difference in mean scores between the two groups was 0.85. This exceeded the calculated MCID thresholds for both the tourniquet (0.51) and non-tourniquet (0.23) groups, suggesting that the between-group difference at this point was not only statistically significant (p<0.05) but also of clinical relevance. These findings support the interpretation that, by the six-week mark, patients in the non-tourniquet group experienced a greater level of recovery that is likely to be meaningful from a patient perspective. This is consistent with numerous previous studies [19-21]. According to the study by Tibrewal et al., patients who had arthroscopic operations without tourniquets had significantly lower pain and operating time. Mean pain was 4.5 in the tourniquet group and 3.58 in the non-tourniquet group postoperatively [19].

A study done by Furia et al. demonstrated reduced analgesic use in the non-tourniquet group postoperatively, with the mean analgesic consumption score being 12.1 in the tourniquet group and 10.2 in the non-tourniquet group. This distinction was statistically significant [22]. While the difference in postoperative opioid consumption between the groups did not reach statistical significance (p=0.069) in our study, the trend towards lower usage in the non-tourniquet group may suggest a clinically relevant effect that warrants further investigation in larger, adequately powered studies.

A meta-analysis of multiple RCTs done by Kuo et al. demonstrated no significant disadvantages or extended operative time when the tourniquet was omitted during KA. There were no significant differences between tourniquet and non-tourniquet groups in postoperative pain, morphine consumption, operative time, or muscle strength [23]. Our results agree with earlier studies by Tibrewal et al. and Furia et al., both of which indicated that using a tourniquet may lead to greater postoperative discomfort or suggested alternative approaches for maintaining a clear surgical field. However, these findings differ from those of Kuo et al., whose meta-analysis concluded that the presence or absence of a tourniquet during ACL reconstruction had no notable effect on postoperative pain or the need for analgesia. Several factors may help explain this divergence. Tourniquet use varied considerably across the studies included in the meta-analysis, with differences in application time, pressure levels, and whether the tourniquet was used continuously or intermittently. In contrast, Tibrewal et al. and Furia et al. followed more uniform or clearly defined protocols. Furthermore, variability in patient characteristics such as age, body mass index, or individual pain thresholds was not consistently accounted for, which may have influenced pain perception. Surgical methods and pain management strategies also likely differed between studies and across time, further complicating direct comparisons. The broader scope and diversity of methodologies in Kuo et al.'s analysis may have reduced the ability to detect more nuanced, yet clinically meaningful, differences in postoperative pain. More recent meta-analyses showed an increase in postoperative pain and no significant extension in operative time in the absence of a tourniquet [24-25]. Wang et al. suggested that absence of tourniquet showed no disadvantage in knee arthroscopy and that the current evidence was more inclined not to use tourniquet as a routine procedure during the KA [26].

Several explanations have been offered regarding the mechanism behind increased pain after the use of a tourniquet in surgery. The use of a tourniquet during surgery triggers a series of ischaemic, inflammatory, and neural changes that contribute to increased postoperative pain. Prolonged occlusion leads to tissue hypoxia and the accumulation of metabolic byproducts, while sudden reperfusion after deflation causes a surge of reactive oxygen species and inflammatory mediators within the tissues [27-28]. These processes result in increased vascular permeability, tissue oedema, and the activation of pain receptors. Wu et al. reported that extended tourniquet times were associated with higher levels of serum creatine kinase, a marker of muscle damage, and greater postoperative pain scores, highlighting the role of ischemic muscle injury [27]. In addition to ischaemic effects, direct mechanical compression by the tourniquet cuff can cause temporary nerve injury, with studies indicating that up to 71% of patients demonstrate transient electromyographic changes after tourniquet use [29]

Owing to the increase in KA surgeries and related tourniquet issues, many approaches to reducing blood loss during and after surgery have been studied. A successful alternative to the tourniquet during a KA is to offer an intravenous sedative along with a local anaesthetic solution including bupivacaine, lidocaine, and adrenaline. It has been shown that this method reduces the need for postoperative painkillers [22]. Another study by Zhang et al. also indicates that insufficient evidence exists to demonstrate the benefits of regular tourniquet usage for patients, as it has not been shown that a tourniquet is helpful for arthroscopic procedures [30].

The study had some limitations. Firstly, the small sample size and observational nature of our study can limit the generalisability of the findings. Secondly, the follow-up period was short and limited to six months, which prevented the assessment of any long-term impact of tourniquet use. Thirdly, the gender imbalance, with a predominance of male participants (82%), could act as a potential confounding factor influencing pain perception and analgesic requirements. The gender distribution reflected the natural presentation of eligible patients during the study period rather than predefined selection criteria. Finally, the lack of assessor blinding could have increased the risk of measurement and performance bias.

While this study provides valuable insight into the impact of tourniquet on postoperative pain, recovery time and opioid analgesic use in knee arthroscopy, future studies should be done to determine the benefits of performing knee arthroscopy with or without tourniquet. The long-term functional outcomes, as well as complications such as delayed healing, should also be investigated to provide a comprehensive understanding of the safety of tourniquet use. Furthermore, different tourniquet pressures can be trialled and compared with no tourniquet use to assess the impact on surgical field visibility, analgesia requirement and recovery time. High-quality RCTs are needed to further clarify the tourniquet use in knee arthroscopy.

## Conclusions

This study indicates that KA surgery performed without the use of a tourniquet does not exhibit any significant drawbacks when compared to procedures involving tourniquet use. Knee arthroscopy without the use of a tourniquet offers the short-term benefit of reducing postoperative pain. Future studies should aim to include a large sample size, blinding of participants, researchers and clinicians who provide follow-up to these patients in clinics and evaluate long-term functional outcomes for a better understanding of the topic.

## References

[1] Onyema C, Oragui E, White J, Khan WS (2011). Evidence-based practice in arthroscopic knee surgery. J Perioper Pract.

[2] Hagino T, Ochiai S, Watanabe Y (2014). Complications after arthroscopic knee surgery. Arch Orthop Trauma Surg.

[3] Liu PL, Li DQ, Zhang YK, Lu QS, Ma L, Bao XZ, Zhang M (2017). Effects of unilateral tourniquet used in patients undergoing simultaneous bilateral total knee arthroplasty. Orthop Surg.

[4] Désiron Q (2007). History of instrumental haemostasis and the particular contribution of Jules E. Péan. Acta Chir Belg.

[5] Kumar Gupta P, Khanna V, Acharya A (2021). Visualization in arthroscopic meniscectomy - portal-site injection versus tourniquet inflation: a prospective, double-blinded, randomised controlled study. J Orthop.

[6] Tsarouhas A, Hantes ME, Tsougias G, Dailiana Z, Malizos KN (2012). Tourniquet use does not affect rehabilitation, return to activities, and muscle damage after arthroscopic meniscectomy: a prospective randomized clinical study. Arthroscopy.

[7] George HL, Kumar G, Mereddy PKR, Harvey RA (20081). Is pneumatic tourniquet necessary in knee arthroscopy. Orthop Procs.

[8] Ostman B, Michaelsson K, Rahme H, Hillered L (2004). Tourniquet-induced ischemia and reperfusion in human skeletal muscle. Clin Orthop Relat Res.

[9] Nagashima M, Otani T, Takeshima K (2020). Unexpectedly high incidence of venous thromboembolism after arthroscopic anterior cruciate ligament reconstruction: Prospective, observational study. J ISAKOS.

[10] Reda W, ElGuindy AM, Zahry G, Faggal MS, Karim MA (2016). Anterior cruciate ligament reconstruction; is a tourniquet necessary? A randomized controlled trial. Knee Surg Sports Traumatol Arthrosc.

[11] Mingo-Robinet J, Castañeda-Cabrero C, Alvarez V, León Alonso-Cortés JM, Monge-Casares E (2013). Tourniquet-related iatrogenic femoral nerve palsy after knee surgery: case report and review of the literature. Case Rep Orthop.

[12] Daniel DM, Lumkong G, Stone ML, Pedowitz RA (1995). Effects of tourniquet use in anterior cruciate ligament reconstruction. Arthroscopy.

[13] Thorblad J, Ekstrand J, Hamberg P, Gillquist J (1985). Muscle rehabilitation after arthroscopic meniscectomy with or without tourniquet control. A preliminary randomized study. Am J Sports Med.

[14] Hooper J, Rosaeg OP, Krepski B, Johnson DH (1999). Tourniquet inflation during arthroscopic knee ligament surgery does not increase postoperative pain. Can J Anaesth.

[15] Hoogeslag RA, Brouwer RW, van Raay JJ (2010). The value of tourniquet use for visibility during arthroscopy of the knee: a double-blind, randomized controlled trial. Arthroscopy.

[16] Nagashima M, Sasaki R, Tanaka K, Takeshima K (2023). The use of tourniquet is useful in terms of blood loss and soft tissue damage in arthroscopic anterior cruciate ligament reconstruction: a retrospective study. Sci Rep.

[17] Júnior LH, Soares LF, Gonçalves MB (2010). Tourniquet versus no tourniquet use in knee videoarthroscopy: a multicentric, prospective, double-blind, randomized clinical trial. Rev Bras Ortop.

[18] Choudhary A, Kanodia N, Agrawal S, Bhasin VB, Singh A (2021). Tourniquet use in arthroscopic ACL reconstruction: a blinded randomized trial. Indian J Orthop.

[19] Tibrewal SB (2001). The pneumatic tourniquet in arthroscopic surgery of the knee. Int Orthop.

[20] Karaoglu S, Dogru K, Kabak S, Inan M, Halici M (2002). Effects of epinephrine in local anesthetic mixtures on hemodynamics and view quality during knee arthroscopy. Knee Surg Sports Traumatol Arthrosc.

[21] Ajnin S, Fernandes R (2020). Reduced length of stay and faster recovery after total knee arthroplasty without the use of tourniquet. J Clin Orthop Trauma.

[22] Furia JP, Zambetti GJ Jr (1992). An injection technique to create a bloodless field in arthroscopically assisted anterior cruciate ligament reconstruction. Am J Sports Med.

[23] Kuo LT, Yu PA, Chen CL, Hsu WH, Chi CC (2017). Tourniquet use in arthroscopic anterior cruciate ligament reconstruction: a systematic review and meta-analysis of randomised controlled trials. BMC Musculoskelet Disord.

[24] Yang Y, Zhang MQ, Zhang JM, Zhang W, Zhou XB, Wang D, Tung TH (2024). Tourniquet use in anterior cruciate ligament reconstruction may decrease operative time but is associated with muscular injury and postoperative pain: a systematic review and meta-analysis of randomized controlled trials. Arthroscopy.

[25] Samei M, Daliri M, Sadeghi M, Ganji R, Parsa A, Ebrahimzadeh MH (2024). Arthroscopic anterior cruciate ligament reconstruction with and without tourniquet use: an updated systematic review and meta-analysis on clinical outcomes. BMC Musculoskelet Disord.

[26] Wang J, Xu W, Lv J (2020). Is it better to routinely use tourniquet for knee arthroscopic surgery: a systematic review and meta-analysis. J Knee Surg.

[27] Wang K, Ni S, Li Z (2017). The effects of tourniquet use in total knee arthroplasty: a randomized, controlled trial. Knee Surg Sports Traumatol Arthrosc.

[28] Zhang M, Liu Q, Meng H (2024). Ischemia-reperfusion injury: molecular mechanisms and therapeutic targets. Signal Transduct Target Ther.

[29] Noordin S, McEwen JA, Kragh JF Jr, Eisen A, Masri BA (2009). Surgical tourniquets in orthopaedics. J Bone Joint Surg Am.

[30] Zhang Y, Li L, Wang J, Li ZH, Shi ZJ (2013). Do patients benefit from tourniquet in arthroscopic surgeries of the knee?. Knee Surg Sports Traumatol Arthrosc.

